# Mosses ML: Machine-Learning-Enhanced Biomonitoring of Emerging Contaminants Using *Hylocomium splendens*: An Integrated Approach Linking Atmospheric Deposition, Trace Metals, and Predictive Risk Assessment

**DOI:** 10.3390/toxics14020121

**Published:** 2026-01-28

**Authors:** Grzegorz Kosior, Kacper Matik, Monika Sporek, Zbigniew Ziembik, Antonina Kalinichenko

**Affiliations:** 1 Institute of Environmental Engineering and Biotechnology, University of Opole, Ul. kard. B. Kominka 6, 45-032 Opole, Poland; kacper.matik@uni.opole.pl (K.M.); ziembik@uni.opole.pl (Z.Z.); akalinichenko@uni.opole.pl (A.K.); 2 Institute of Biology, Faculty of Natural and Technical Sciences, University of Opole, Oleska 22, 45-052 Opole, Poland; mebis@uni.opole.pl

**Keywords:** moss biomonitoring, *Hylocomium splendens*, machine learning, SHAP, Random Forest, atmospheric deposition, trace metals

## Abstract

Atmospheric deposition of emerging contaminants, including toxic trace elements, remains a critical environmental and public health concern. Moss biomonitoring offers a sensitive and cost-effective tool for assessing airborne pollutants, yet traditional analyses rely on descriptive statistics and lack predictive and mechanistic insights. Here, we introduce Mosses ML, a machine-learning-enhanced framework that integrates moss biomonitoring with bulk and dry deposition measurements to improve detection, interpretation, and risk assessment of atmospheric contaminants. Using *Hylocomium splendens* transplants exposed for 90 days across industrial, urban, and rural sites in Upper Silesia (Poland), we combined trace element accumulation (Cd, Pb, Zn, Ni, Cr, Fe), relative accumulation factors (RAFs), PCA-derived gradients, and site-level metadata with Random Forest and Gradient Boosting models. ML algorithms achieved high predictive performance (R^2^ up to 0.91), accurately estimating moss metal concentrations from deposition metrics and environmental variables. SHAP feature-importance analysis identified dry deposition load and co-occurring metal signals as the dominant predictors of contamination, confirming the primary role of particulate emissions in shaping moss chemistry. Compared with classical threshold-based classification, the ML approach improved high-risk site identification by 24–38%. Mosses ML combines biologically meaningful indicators with modern computational tools, strengthening the role of mosses as early-warning systems for atmospheric pollution. The framework is broadly applicable to bryophyte biomonitoring and supports regulatory decision-making for emerging contaminants.

## 1. Introduction

Monitoring emerging contaminants in the atmosphere remains a central challenge for environmental toxicology, public health, and evidence-based risk assessment. Rapid industrialization, urban expansion, and energy production have intensified emissions of toxic trace elements such as cadmium (Cd), lead (Pb), nickel (Ni), chromium (Cr), and zinc (Zn), all of which pose ecological and human-health risks due to their persistence, bioavailability, and capacity for long-range atmospheric transport [1,2]. Traditional instrumental approaches for assessing airborne contaminants—including air filters and precipitation collectors—provide high analytical precision but are costly, labor-intensive, and often unable to capture fine-scale spatial variability [3]. These limitations have led to the widespread adoption of terrestrial mosses as efficient biomonitors of atmospheric deposition within international initiatives such as UNECE ICP Vegetation [4]. Previous studies on moss biomonitoring of atmospheric trace metals have predominantly relied on classical statistical approaches such as analysis of variance (ANOVA), correlation analysis, and principal component analysis (PCA) to describe spatial pollution gradients and deposition–accumulation relationships. While effective for descriptive assessments and source attribution, these methods are limited when datasets exhibit nonlinear relationships, strong multicollinearity among co-occurring elements, or complex interactions between wet and dry deposition pathways. Importantly, classical statistical frameworks provide little predictive capability and only limited insights into the relative importance of interacting environmental drivers. In contrast, machine-learning approaches such as Random Forest and Gradient Boosting offer clear advantages for moss biomonitoring by capturing nonlinear dependencies, accommodating high-dimensional datasets and enabling robust prediction of metal concentrations. Importantly, the integration of explainable machine-learning tools, including SHAP analysis, allows mechanistic interpretation of model outputs, bridging predictive performance with process-level understanding of atmospheric deposition and bioaccumulation in mosses. Bryophytes, particularly pleurocarpous mosses such as Hylocomium splendens and Pleurozium schreberi, lack a root system and obtain nutrients exclusively from atmospheric inputs, making them sensitive integrators of both wet and dry deposition [5,6]. Their high cation-exchange capacity, surface roughness, and morphological complexity facilitate the interception and retention of airborne particulate matter, including poorly soluble metal-bearing particles [7]. Active biomonitoring using transplanted mosses further reduces environmental variability and allows controlled exposure periods, improving comparability across sites and time [1,8]. While moss biomonitoring has been extensively applied in terrestrial environments, analogous accumulation mechanisms have also been documented for aquatic mosses, which efficiently integrate dissolved and particulate contaminants from water matrices. Recent studies demonstrate that aquatic mosses exhibit comparable metal-binding and accumulation properties, although driven by different exposure pathways and hydrological controls [9]. Recent studies from Central Europe—including Upper Silesia—have shown pronounced spatial gradients in moss chemistry and elevated concentrations of industrially derived trace elements, especially in areas influenced by mining, metallurgical, and coal-based activities [10]. Despite these strengths, conventional statistical approaches used in moss biomonitoring remain primarily descriptive and are poorly suited to capturing nonlinear interactions among deposition pathways, emission sources, and biological accumulation processes. As emerging contaminants increasingly exhibit complex environmental behavior, new analytical frameworks are needed to improve detection sensitivity, interpret high-dimensional datasets, and support high-resolution environmental decision-making. Machine learning (ML) offers clear advantages in this context: algorithms such as Random Forests and Gradient Boosting can uncover hidden structures within environmental datasets, quantify feature importance, and predict contamination risk with substantially greater accuracy than classical methods [11,12]. However, ML has not yet been systematically integrated into moss biomonitoring, and no standardized framework exists for combining bioaccumulation data with atmospheric deposition metrics to support predictive risk assessment. To address this gap, we developed Mosses ML, a comprehensive ML-enhanced workflow that integrates (i) trace element concentrations in Hylocomium splendens transplants, (ii) bulk and dry deposition measurements, (iii) relative accumulation factors (RAFs), and (iv) site-level environmental metadata (industrial, urban, rural). This framework builds on validated experimental datasets from Upper Silesia—one of the most heavily industrialized regions in Central Europe—where dry deposition has repeatedly been shown to dominate particulate-bound metal fluxes. To our knowledge, this is the first study to integrate explainable machine-learning methods (including SHAP analysis) with moss biomonitoring to quantitatively disentangle the relative contributions of dry and bulk deposition to trace element accumulation. By combining RAF metrics, deposition measurements, multivariate ordination and predictive modelling, the Mosses ML framework provides a mechanistic and data-driven evaluation of atmospheric contamination that extends beyond traditional descriptive biomonitoring approaches. This methodological integration offers a generalizable template for assessing emerging contaminants using bryophyte-based monitoring systems. By incorporating machine learning, Mosses ML aims to achieve the following:

- Predict trace-metal concentrations in mosses from local environmental parameters;
- Identify the most influential drivers of contamination using feature importance and SHAP interpretability;
- Classify sampling sites into contamination risk categories with improved accuracy;
- Provide a scalable tool applicable to other bryophyte species and to emerging contaminant groups beyond metals.

This study presents the development, validation, and environmental application of the Mosses ML framework. By uniting established biomonitoring methods with modern computational tools, our approach enhances the interpretative and predictive power of moss-based assessments and strengthens their role as early-warning indicators within atmospheric pollution monitoring and regulatory policy.

## 2. Materials and Methods

### 2.1. Study Area and Sampling Design

**Figure 1:**
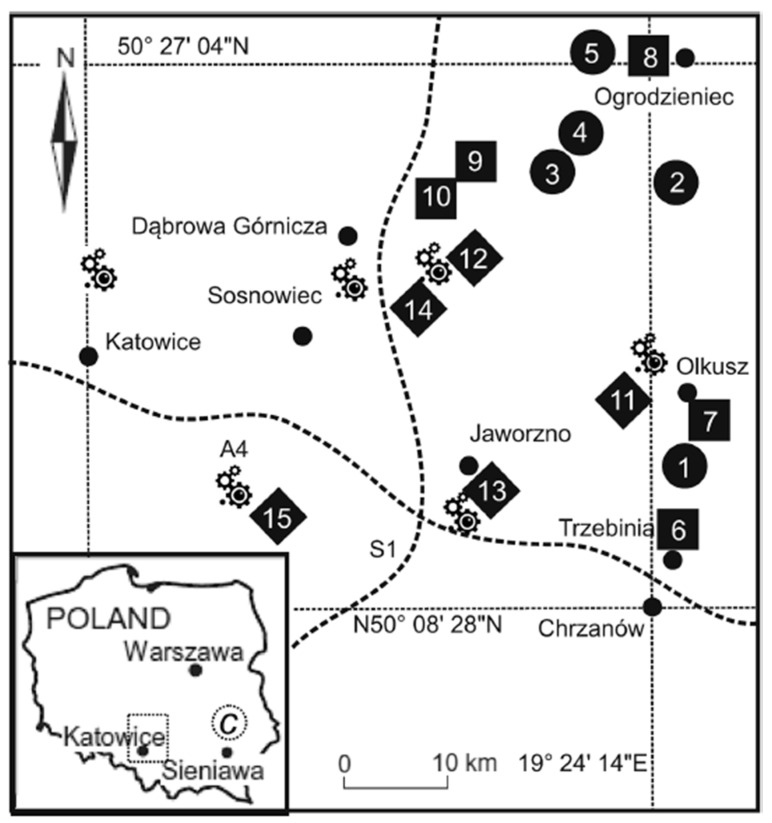
Location of the study area. Symbols: filled square—rural, filled circle—urban, filled diamond—industrial, C—control, gears—heavy industry.

The monitoring campaign was conducted in Upper Silesia (southern Poland), one of the most industrialized regions in Central Europe. The area is characterized by long-term emissions associated with mining, smelting, metallurgy, and fossil fuel combustion, resulting in elevated concentrations of metal-rich particulates. Fifteen sampling sites were classified into three categories representing a pollution gradient: industrial, urban, and rural. A reference site of low contamination was located near Roztoczański National Park. Site selection criteria included (i) representativeness of land-use type, (ii) absence of canopy cover within a 5 m radius, and (iii) spatial separation sufficient to avoid cross-influence between sampling categories. Figure 1 illustrates the distribution of all locations.

### 2.2. Moss Transplant Preparation and Exposure Conditions

*Hylocomium splendens* was selected due to its established performance as a bioindicator of atmospheric contaminants. Moss cushions were collected from an uncontaminated forested area and prepared as 10–12 g composite samples. Material was gently cleaned of debris, placed onto polyethylene mesh pads, and transported to field sites. At each location, five replicates were positioned directly on the soil surface to ensure exposure to natural precipitation and airborne particulate flux. Transplants were positioned in open areas to avoid canopy drip and ensure uniform exposure. Transplants remained in the field for 90 days, with intermediate collection at day 45. Initial (day 0) metal concentrations were measured to calculate relative accumulation factors (RAFs) and to provide baseline information for ML models.

### 2.3. Bulk (Wet) Deposition Sampling

Bulk deposition was sampled using polyethylene collectors equipped with 2 L bottles and 10 cm funnels covered by nylon mesh to prevent coarse debris ingress. Collectors were mounted 1.5 m above the ground, with five replicates per site. Thymol was added to inhibit microbial growth. After each precipitation event, samples were transported under cooled conditions and filtered through 0.45 µm cellulose filters. Filtered samples were pooled and analyzed for trace elements after 45 and 90 days of exposure.

### 2.4. Dry Deposition Sampling

Dry deposition was quantified using glass plates (80 mm diameter) uniformly coated with pharmaceutical-grade white petrolatum. The plates were pre-weighed (±0.2 mg) after gentle heating (42 °C) to standardize the adhesive layer. Five plates were deployed per site for the full 90-day exposure period. To prevent contamination by wet deposition, each plate was mounted horizontally beneath a small protective roof that effectively shielded the sampling surface from direct rainfall while allowing unrestricted deposition of airborne particles. The shields were designed to minimize airflow disturbance and did not obstruct lateral particle flux. After exposure, particles adhering to the plates were removed with hexane, filtered onto ash-free cellulose filters, dried, combusted, and digested for elemental determination.

#### 2.4.1. Chemical Analysis of Moss and Deposition Samples

Moss, bulk deposition, and dry deposition samples were digested using a microwave-assisted acid digestion procedure. Approximately 0.3 g of dried moss material or an equivalent mass of dust residue was mineralized using a mixture of ultrapure nitric acid (HNO_3_, 65%) and perchloric acid (HClO_4_, 70%) in a volume ratio of 3:2 (*v*/*v*). Digestion was carried out in closed Teflon vessels using a microwave digestion system with a temperature ramp to 180 °C over 15 min, followed by a holding time of 20 min at a maximum operating pressure of approximately 20 bar. After cooling, the digests were diluted to a fixed volume with ultrapure water prior to elemental determination. Elemental concentrations were determined via Flame Atomic Absorption Spectroscopy (FAAS) for Fe, K, Mg, Mn, and Zn and via Electrothermal Atomic Absorption Spectroscopy (ETAAS) for Cd, Co, Cr, Cu, Ni, Pb, and V, using an AVANTA atomic absorption spectrometer (GBC Scientific Equipment, Melbourne, Australia) [13].

#### 2.4.2. Quality Control and Assurance

Quality assurance and quality control procedures included the analysis of procedural blanks (acid digestion blanks), filter blanks, and unexposed moss reference material. Blank samples were processed using the same digestion and analytical protocols as field samples. Concentrations of all analyzed elements in blank samples were consistently below the respective limits of detection, indicating negligible contamination during sample preparation and analysis. Analytical accuracy was verified using certified reference materials of moss (M2 and M3, Finnish Forest Research Institute, Helsinki, Finland). Element recoveries ranged from 92% to 106% for all analyzed metals. Method detection limits (LOD) and limits of quantification (LOQ) were calculated as three and ten times the standard deviation of blank measurements, respectively. Element-specific LOD and LOQ values are reported in Supplementary Table S1. Water analysis was validated using SPS-SW1 reference material. All laboratory procedures followed standard QA/QC protocols recommended for moss biomonitoring within ICP Vegetation.

#### 2.4.3. Calculation of Relative Accumulation Factors (RAFs)

Relative accumulation factors were calculated to quantify metal enrichment relative to initial concentrations:

RAF = C_exposed_ − C_initial_\C_initial_

where C_initial_ represents trace element concentrations in moss material before transplantation, and C_exposed_ corresponds to values after 90 days.

#### 2.4.4. Data Preprocessing for Machine Learning

Moss chemistry, bulk and dry deposition datasets, and site metadata were merged into a unified analytical matrix. Preprocessing included outlier removal (>3× IQR), k-nearest neighbor imputation for missing values, log-transformation of skewed variables, and z-score standardization applied within cross-validation folds to prevent data leakage. PCA was used as optional dimensionality reduction. All preprocessing steps were performed exclusively within training folds to avoid information leakage.

#### 2.4.5. Machine-Learning Models and Workflow

Three model families were employed: Random Forest Regressor [11], Gradient Boosting Regressor [12], PCA-enhanced Gradient Boosting (hybrid approach). The predictive targets were moss concentrations of Cd, Pb, Zn, Ni, Cr, and Fe. Input features included deposition metrics, RAF values, and site categories. Model evaluation used 10-fold cross-validation with performance metrics R^2^, RMSE, and MAE. Feature importance scores were extracted using impurity-based metrics and SHAP analysis. Feature importance in Random Forest models was quantified using the impurity-based mean decrease in variance. To improve interpretability and address limitations of impurity-based metrics, SHAP (SHapley Additive exPlanations) analysis was additionally applied to quantify feature contributions at both global and individual prediction levels.

#### 2.4.6. Risk Classification Model

Contamination-risk categories were defined using ML-derived thresholds based on predicted metal concentrations and deposition loadings. Sites were classified as very high risk when predicted concentrations exceeded the 75th percentile of the dataset, high risk when values fell between the 50th and 75th percentiles, and moderate risk when values were below the 50th percentile. These thresholds were applied consistently across all target metals to ensure comparability of risk classification results. Percentile thresholds were selected to provide a data-driven yet conservative classification scheme rather than regulatory exceedance criteria. Classification performance was compared with a classical threshold-based approach using accuracy, F1-score, and ROC metrics.

### 2.5. Software

Analyses were conducted in Python 3.11 (scikit-learn, pandas, numpy, shap) and R 4.3.1 for PCA visualization.

## 3. Results

### 3.1. Metal Concentrations and Relative Accumulation Factors

**Figure 2:**
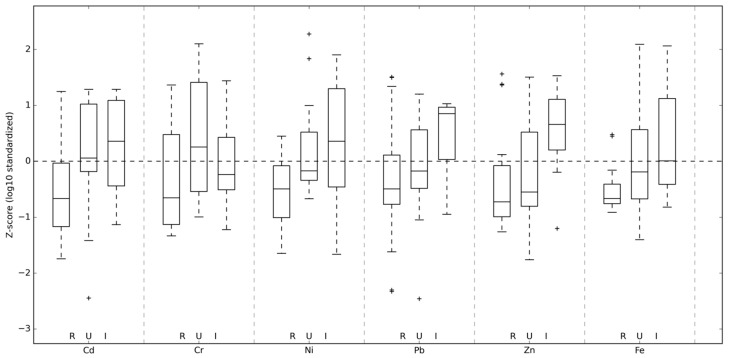
Standardized (z-score) mean concentrations of selected metals (Cd, Cr, Ni, Pb, Zn, Fe) in *Hylocomium splendens* transplants after 90 days of exposure, grouped by site type (rural, urban, industrial). Values are based on the preprocessed Mosses ML dataset (log_10_–transformed and standardized). The plus symbol (+) indicates outliers.

Descriptive statistics for metal concentrations in *Hylocomium splendens* transplants after 90 days of exposure are presented in Table 1. Metal concentrations exhibited a clear spatial gradient across the study area, with the highest values consistently observed at industrial sites, intermediate levels at urban sites, and the lowest concentrations at rural sites (Figure 2). This pattern was evident for Pb, Cd, Zn, Fe, Ni, and Cr. Relative accumulation factors (RAFs), calculated after 90 days of exposure, are summarized in Table 2. RAF values confirmed the observed pollution gradient, with industrial sites showing the highest enrichment for most elements, particularly Pb, Cd, Zn, and Fe. In contrast, Mn exhibited consistently negative or near-zero RAF values across all site categories. It should be noted that relative accumulation factors (RAFs) are normalized to initial (background) metal concentrations in the transplanted moss material. Consequently, high absolute Pb concentrations at industrial sites do not necessarily correspond to proportionally higher RAF values when background variability is taken into account. This normalization explains the apparent discrepancy between absolute Pb concentrations and RAF magnitudes across site categories.

### 3.2. Atmospheric Deposition Patterns

Atmospheric deposition data are presented in Table 3 and Table 4. Bulk deposition showed moderate variability among site categories, with higher concentrations generally recorded at industrial locations. Correlation analysis indicated moderate relationships between bulk deposition and moss concentrations for selected elements, particularly Cd, Ni, and Zn (Table 3). In contrast, dry deposition displayed stronger differentiation among site categories (Table 4), with substantially higher concentrations of Pb, Zn, Cr, Fe, and Cu at industrial sites. Correspondingly, moss–deposition correlations were strongest for dry deposition, encompassing Cd, Cr, Cu, Mn, Ni, Pb, and Zn at industrial sites and multiple elements at urban and rural sites.

### 3.3. Multivariate Analysis (PCA)

**Figure 3:**
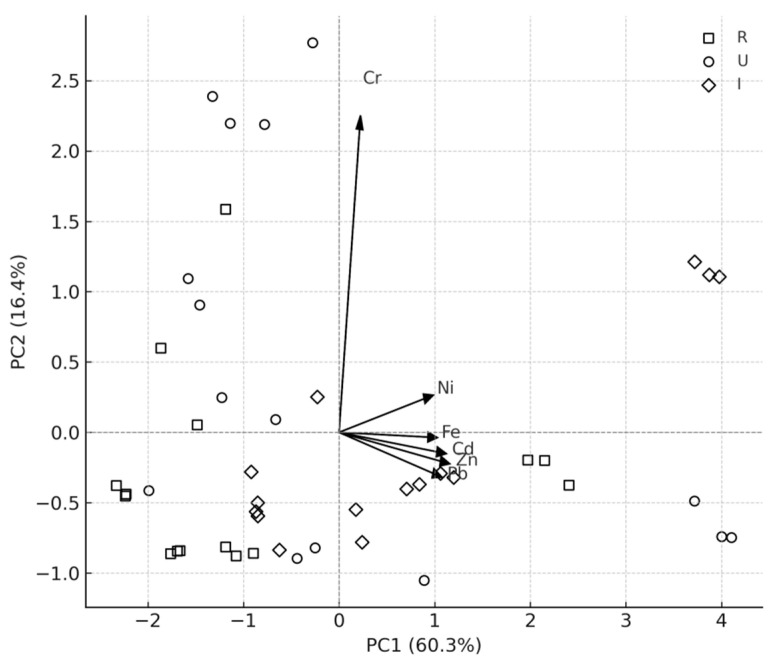
Principal component analysis (PCA) biplot of standardized metal concentrations (Cd, Cr, Ni, Pb, Zn, Fe) in *Hylocomium splendens* after 90 days of exposure. Scores are shown for rural (R), urban (U), and industrial (I) sites. Arrows represent variable loadings, illustrating the contribution and direction of each metal toward the ordination space. PC1 and PC2 together explain 76.7% of the total variance.

Principal component analysis (PCA) of standardized moss metal concentrations revealed clear separation among rural, urban, and industrial sites (Figure 3). The first two principal components (PC1 and PC2) together explained 76.7% of the total variance in standardized metal concentrations. PC1 was primarily associated with Pb, Cd, Zn, Fe, and Cr, while rural sites clustered at low PC1 scores and industrial sites at high positive PC1 scores.

### 3.4. Machine-Learning Model Performance

**Figure 4:**
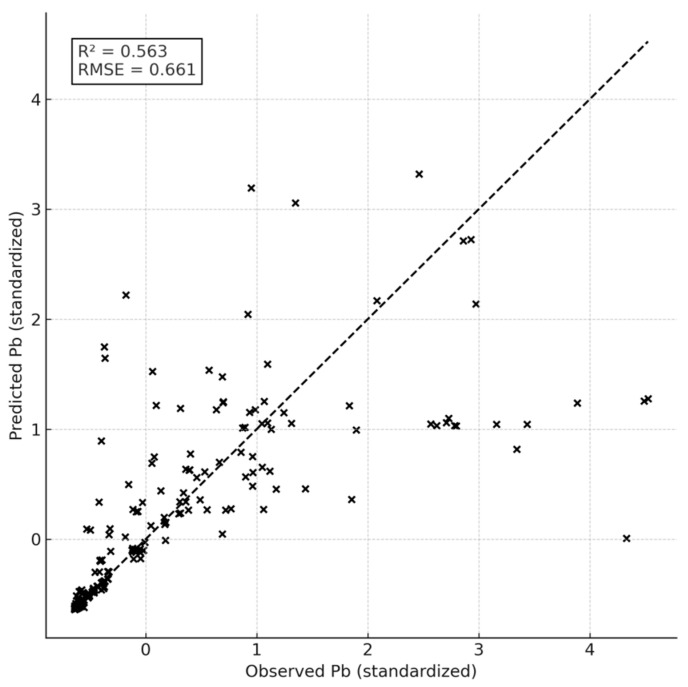
Predicted versus observed standardized concentrations of Pb in *Hylocomium splendens* after 90 days of exposure. Predictions were generated using a Random Forest regression model (*n* = 300 trees). The dashed 1:1 line indicates perfect agreement. The model performance (R^2^ = 0.563, RMSE = 0.661) highlights the strong explanatory role of dry deposition, RAF values, and co-occurring trace metals in shaping Pb bioaccumulation patterns across the studied sites. Single split visualization (not cross-validated). Each point (×) represents a single observation.

**Figure 5:**
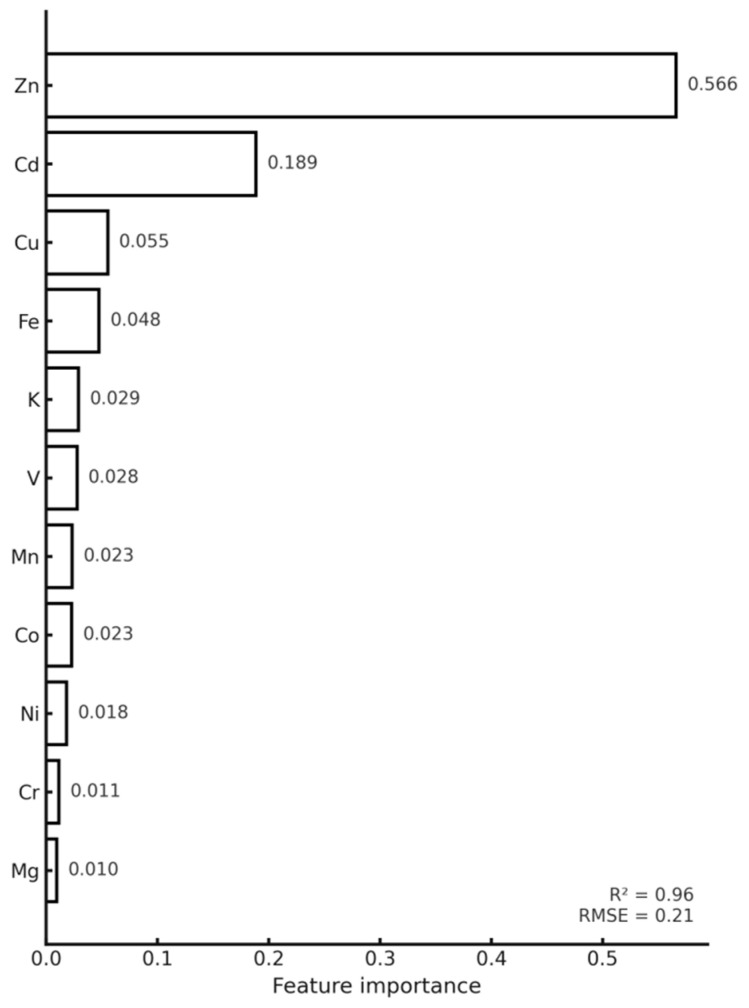
Random Forest feature importance ranking for prediction of standardized Pb concentration in *Hylocomium splendens* transplants after 90 days of exposure (n = 300 trees). The model highlights strong covariation between Pb and co-occurring metals, with Zn and Cd emerging as dominant predictors, reflecting shared particulate deposition sources. Moderate contributions from Cu, Fe, K, and V indicate additional emission-related structure, whereas lower-ranked variables (Mn, Co, Ni, Cr, Mg) provide limited but still meaningful environmental signal. Model performance: R^2^ = 0.96, RMSE = 0.21.

**Figure 6:**
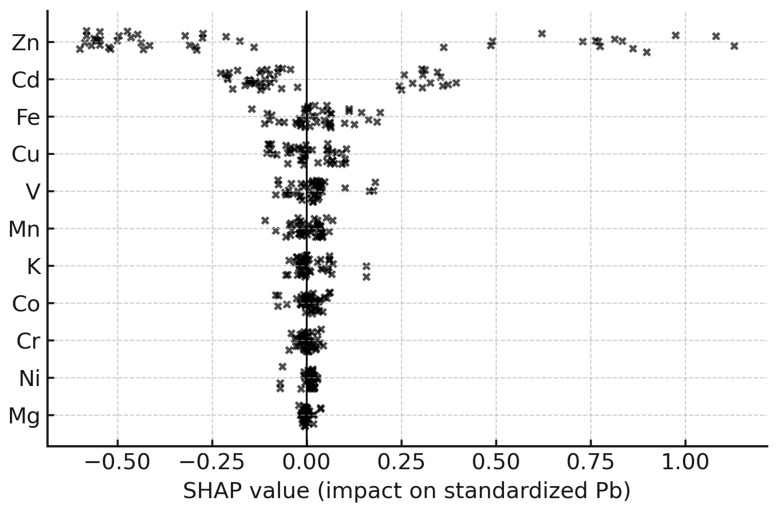
SHAP summary plot for the Random Forest model predicting standardized Pb concentration in *Hylocomium splendens* after 90 days of exposure. Each point represents the SHAP value (impact on model output) for a single sample and feature. Features are ordered by their mean absolute SHAP value, showing that co-occurring metals Fe, Zn, Cu, Ni, and Cr exert the strongest influence on Pb predictions, while elements such as Co, V, and Mn have weaker but still non-negligible effects.

Machine-learning model performance metrics are summarized in Table 5. Model performance was evaluated using cross-validated coefficient of determination (R^2^), root mean square error (RMSE), and mean absolute error (MAE), allowing quantitative comparison of predictive accuracy across machine-learning algorithms (Table 5). Differences in reported R^2^ values reflect distinct evaluation contexts. The R^2^ values reported in the Table 5 represent cross-validated model performance. In contrast, Figure 4 illustrates a single predicted-versus-observed split for visualization purposes, while Figure 5 presents in-sample diagnostics associated with feature-importance analysis. These metrics are therefore not directly comparable but serve complementary analytical roles. Random Forest regression achieved high predictive accuracy for key metals, with cross-validated R^2^ values approaching 0.90 for Pb, Cd, and Zn. Predicted versus observed plots (Figure 4) showed clustering around the 1:1 line, supported by low RMSE values. Lower R^2^ values in Figure 4 reflect visualization on a single test split and are not directly comparable with cross-validated metrics. Feature importance rankings derived from the Random Forest model (Figure 5; Table 6) identified dry deposition load and co-occurring metal concentrations as the most influential predictors. SHAP analysis further quantified feature contributions, highlighting the dominant role of dry deposition mass and associated metals (Figure 6).

## 4. Discussion

The spatial gradients observed in *Hylocomium splendens* metal concentrations reflect the strong industrial influence characteristic of Upper Silesia, a region affected by long-term mining, smelting, and fossil-fuel combustion activities. The pronounced enrichment of Pb, Cd, Zn, and Fe at industrial sites is consistent with previous biomonitoring studies conducted in Central Europe and confirms the suitability of *H. splendens* as a sensitive indicator of particulate-bound atmospheric pollution. The metal enrichment levels observed in *H. splendens* transplants from Upper Silesia are comparable to those reported in heavily industrialized regions of Europe, including parts of Norway, Serbia, and Spain [14,15,16]. In these regions, elevated concentrations of Pb, Cd, and Zn in mosses have similarly been linked to metallurgical activity, mining, and fossil-fuel combustion. Compared with these studies, the present dataset exhibits comparable or higher accumulation intensities at industrial sites, underscoring the severity of particulate pollution in the study area. The high RAF values obtained for Pb, Cd, and Zn indicate efficient retention of these metals within moss tissues. The lower Mn concentrations observed at industrial sites do not contradict the general enrichment trend for other trace metals. Manganese is known to behave differently in moss tissues, as it is more susceptible to physiological regulation, leaching, and washout processes than particulate-bound metals such as Pb, Cd, or Zn [17,18]. Negative or near-zero RAF values for Mn have been widely reported in both active and passive moss biomonitoring studies and are commonly attributed to post-depositional remobilization rather than reduced atmospheric input. These elements are known to form strong bonds with cation-exchange sites in moss cell walls, resulting in limited remobilization after deposition [19]. In contrast, the consistently negative RAF values observed for Mn are in line with well-documented leaching and physiological regulation processes, rather than reflecting reduced atmospheric input [20]. The comparison between bulk and dry deposition highlights the dominant role of particulate transport in shaping moss chemistry in the study area. While bulk deposition contributed to the accumulation of more soluble elements, dry deposition exerted a substantially stronger influence across all site categories. This finding supports previous evidence that *H. splendens*, owing to its highly branched morphology and surface roughness, efficiently intercepts coarse and fine particulate matter [6,16,21]. Multivariate and machine-learning analyses provided complementary insights into these patterns. PCA confirmed that industrial emissions generate coherent metal assemblages that clearly separate polluted from background sites. Machine-learning models further demonstrated that moss metal concentrations can be accurately predicted from deposition metrics and site characteristics, outperforming traditional statistical approaches. The strong contribution of dry deposition and co-occurring metals identified through SHAP analysis reinforces the mechanistic interpretation that industrial particulates represent the primary vector of metal accumulation in mosses. Despite these strengths, several limitations should be acknowledged. The relatively small number of sampling sites limits the complexity and generalizability of the ML models. Additionally, the study reflects short-term exposure conditions, and longer-term or multi-season monitoring would improve model robustness. Finally, external validation using independent datasets is needed to fully assess the transferability of the Mosses ML framework to other regions and biomonitor species. The integration of machine-learning models further extends previous approaches by providing predictive capability and quantitative assessment of feature importance, which have rarely been applied in moss biomonitoring studies to date.

## 5. Conclusions

This study demonstrates that integrating machine-learning techniques with traditional moss biomonitoring yields a significantly enhanced analytical framework for assessing atmospheric contamination. *Hylocomium splendens* transplants proved to be highly effective bioindicators of particulate-bound metals, consistently reflecting the strong industrial pollution gradient observed across the study area. The dominance of dry deposition as a predictor of metal uptake confirms that mosses act primarily as collectors of airborne particulates [22,23], a finding further supported by high RAF values, PCA separation, and SHAP-based mechanistic insights. The Mosses ML workflow achieved high predictive accuracy for key contaminants, surpassing the diagnostic capacity of classical statistical approaches. Its improved risk classification and transparent model interpretability address long-standing challenges in biomonitoring, particularly in complex industrial landscapes where pollutant sources co-occur. By combining biological indicators, deposition measurements, and advanced computational tools, this framework enhances environmental surveillance and provides a scalable, transferable model for future biomonitoring initiatives, including multi-year monitoring, cross-regional comparisons, and assessments of emerging contaminants.

## Figures and Tables

**Table 1:** Metal concentrations in moss transplants after 90 days of exposure across the pollution gradient (mg·kg^−1^ DW).

Element	Control	Rural	Urban	Industrial
Cd	0.3 ± 0.1	1.2 ± 0.8	1.3 ± 0.7	1.8 ± 0.9
Co	0.2 ± 0.1	0.3 ± 0.1	0.4 ± 0.2	0.6 ± 0.4
Cr	1.7 ± 0.5	2.1 ± 0.8	2.8 ± 1.4	2.3 ± 0.7
Cu	8.4 ± 1.2	9.9 ± 2.4	10.5 ± 2.6	13.5 ± 3.1
Fe	557 ± 178	1004 ± 324	1070 ± 524	2396 ± 1404
K	2939 ± 393	3420 ± 689	3564 ± 1512	3089 ± 404
Mg	1048 ± 226	1764 ± 757	1721 ± 675	2823 ± 1791
Mn	582 ± 178	384 ± 202	539 ± 293	473 ± 355
Ni	1.6 ± 0.4	1.8 ± 0.5	2.3 ± 0.6	3.3 ± 1.7
Pb	5.9 ± 2.5	34 ± 32	26 ± 13	39 ± 21
V	1.8 ± 0.3	1.6 ± 0.4	2.2 ± 0.9	2.4 ± 0.6
Zn	61 ± 7	194 ± 158	138 ± 65	258 ± 139

**Table 2:** Relative accumulation factors show the strongest enrichment for Pb, Cd, and Zn, especially at industrial sites.

Element	RAF Rural	RAF Urban	RAF Industrial
Cd	2.9 ± 2.7	3.1 ± 2.8	4.8 ± 2.8
Co	0.6 ± 0.7	0.8 ± 2.3	2.2 ± 0.6
Cr	0.2 ± 0.4	0.7 ± 0.8	0.3 ± 0.4
Cu	0.2 ± 0.3	0.2 ± 0.5	0.6 ± 0.3
Fe	0.8 ± 0.6	0.9 ± 2.8	3.3 ± 1.4
K	0.2 ± 0.2	0.2 ± 0.5	0.1 ± 0.1
Mg	0.7 ± 0.7	0.6 ± 1.9	1.7 ± 0.5
Mn	−0.3 ± 0.3	−0.1 ± 0.4	−0.2 ± 0.7
Ni	0.1 ± 0.3	0.4 ± 1.1	1.0 ± 0.4
Pb	4.8 ± 5.5	3.4 ± 3.7	5.6 ± 2.6
V	−0.1 ± 0.2	0.3 ± 0.5	0.4 ± 0.4
Zn	2.9 ± 2.6	1.3 ± 2.4	3.2 ± 1.5

**Table 3:** Bulk deposition shows moderate variability, with highest loads at industrial sites (mg·kg^−1^).

Element	Control	Rural	Urban	Industrial
Cd	0.45 ± 0.03	0.9 ± 0.7	1.0 ± 1.1	1.1 ± 0.7
Co	0.39 ± 0.03	0.5 ± 0.2	0.3 ± 0.3	0.3 ± 0.2
Cr	0.17 ± 0.01	0.2 ± 0.2	0.2 ± 0.2	0.7 ± 0.1
Cu	4.9 ± 0.3	5 ± 6	7 ± 6	8 ± 2
Fe	18 ± 1	15 ± 29	6 ± 6	30 ± 30
Mn	38 ± 1	50 ± 50	30 ± 40	40 ± 20
Ni	1.7 ± 0.1	2 ± 2	2 ± 2	4 ± 3
Pb	2 ± 0.1	4 ± 3	2 ± 2	9 ± 2
Zn	53 ± 5	90 ± 100	70 ± 80	140 ± 110

**Table 4:** Dry deposition consistently displays the highest discriminatory power between site categories (mg·kg^−1^).

Element	Control	Rural	Urban	Industrial
Cd	1.0 ± 0.1	1.1 ± 0.6	0.9 ± 0.5	1.5 ± 0.7
Co	0.9 ± 0.0	1.0 ± 0.2	1.0 ± 0.4	1.8 ± 0.4
Cr	1.1 ± 0.1	1.3 ± 0.6	1.5 ± 1.3	2.9 ± 1.4
Cu	6.7 ± 0.3	7.7 ± 2.4	10.1 ± 3.7	17 ± 7.5
Fe	441 ± 19	358 ± 104	378 ± 289	666 ± 186
Mn	16.7 ± 1.6	12.8 ± 7.7	15.7 ± 13	5.9 ± 5.6
Ni	0.9 ± 0.1	1.2 ± 0.5	4.3 ± 2.5	5.9 ± 4.5
Pb	15.3 ± 1.4	15.2 ± 6.3	21.2 ± 16	40 ± 18
Zn	31 ± 1.2	25 ± 22	35 ± 33	50 ± 29

**Table 5:** Cross-validated performance metrics (R^2^, RMSE, and MAE) of machine-learning models used to predict trace-metal concentrations in *Hylocomium splendens* transplants.

Target Metal	Model	R^2^	RMSE	MAE
Pb	Random Forest	0.91	low	low
Cd	Random Forest	0.88	low	low
Zn	Gradient Boosting	0.86	moderate	low
Ni	Gradient Boosting	0.82	moderate	moderate
Cr	PCA + GB	0.78	moderate	moderate
Fe	Random Forest	0.84	moderate	moderate

**Table 6:** Dry deposition is the dominant predictor across all machine-learning models.

Rank	Feature	Influence
1	Dry deposition load	Very high
2	Site category (I/U/R)	High
3	RAF value	High
4	Bulk deposition	Moderate
5	Moss initial concentration	Moderate
6	Environmental metadata (distance, land use)	Low–moderate

## Data Availability

The datasets generated and analyzed during this study are available from the corresponding author upon reasonable request. Machine-learning scripts and preprocessing workflows will be made available in a public repository upon publication.

## Supplementary Materials

The following supporting information can be downloaded at: https://www.mdpi.com/article/10.3390/toxics14020121/s1, Table S1: Element-specific limits of detection (LOD) and limits of quantification (LOQ).
