# Supplementary material for: Mosses ML: Machine-Learning-Enhanced Biomonitoring of Emerging Contaminants Using Hylocomium splendens: An Integrated Approach Linking Atmospheric Deposition, Trace Metals, and Predictive Risk Assessment
Source: Toxics. 2026 Jan 28;14(2):121. doi: 10.3390/toxics14020121 (PMC12944864; doi:10.3390/toxics14020121)
Supplement: Supplementary file 1 [file toxics-14-00121-s001.zip › toxics-4066300-supplementary.pdf]

Table S1. Element-specific method detection limits (LOD) and limits of quantification (LOQ) for trace-metal analyses. LOD and LOQ were calculated as 3×SD and 10×SD of blank measurements, respectively. Units: mg·kg^{-1} DW.

**Element**	**Technique (FAAS/ETAAS)**	**SD of blanks (mg·kg^{-1})**	**LOD = 3×SD (mg·kg^{-1})**	**LOQ = 10×SD (mg·kg^{-1})**
Cd	ETAAS	0.001	0.003	0.01
Co	ETAAS/FAAS	0.003	0.009	0.03
Cr	ETAAS/FAAS	0.004	0.012	0.040
Cu	ETAAS/FAAS	0.005	0.015	0.05
Fe	FAAS	0.05	0.15	0.5
Mn	FAAS	0.01	0.03	0.1
Ni	ETAAS/FAAS	0.004	0.012	0.04
Pb	ETAAS	0.003	0.009	0.03
V	ETAAS/FAAS	0.005	0.015	0.05
Zn	FAAS	0.02	0.06	0.2
